# OSlms: A Web Server to Evaluate the Prognostic Value of Genes in Leiomyosarcoma

**DOI:** 10.3389/fonc.2019.00190

**Published:** 2019-03-29

**Authors:** Qiang Wang, Longxiang Xie, Yifang Dang, Xiaoxiao Sun, Tiantian Xie, Jinshuai Guo, Yali Han, Zhongyi Yan, Wan Zhu, Yunlong Wang, Wei Li, Xiangqian Guo

**Affiliations:** ^1^Department of Preventive Medicine, Institute of Biomedical Informatics, Joint National Laboratory for Antibody Drug Engineering, Cell Signal Transduction Laboratory, Bioinformatics Center, School of Software, School of Basic Medical Sciences, Henan University, Kaifeng, China; ^2^Department of Anesthesia, Stanford University, Stanford, CA, United States; ^3^Henan Bioengineering Research Center, Zhengzhou, China; ^4^Division of Biostatistics, Dan L. Duncan Cancer Center Department of Molecular and Cellular Biology Baylor College of Medicine, Houston, TX, United States

**Keywords:** leiomyosarcoma, prognostic, web server, database, survival analysis

## Abstract

The availability of transcriptome data and clinical annotation offers the opportunity to identify prognosis biomarkers in cancer. However, efficient online prognosis analysis tools are still lacking. Herein, we developed a user-friendly web server, namely **O**nline consensus **S**urvival analysis of **l**eio**m**yo**s**arcoma (OSlms), to centralize published gene expression data and clinical datasets of leiomyosarcoma (LMS) patients from The Cancer Genome Atlas (TCGA) and Gene Expression Omnibus (GEO). OSlms comprises of a total of 268 samples from three independent datasets, and employs the Kaplan Meier survival plot with hazard ratio (HR) and log rank test to estimate the prognostic potency of genes of interests for LMS patients. Using OSlms, clinicians and basic researchers could determine the prognostic significance of genes of interests and get opportunities to identify novel potential important molecules for LMS. OSlms is free and publicly accessible at http://bioinfo.henu.edu.cn/LMS/LMSList.jsp.

## Introduction

Leiomyosarcoma (LMS) is a rare malignant soft tissue tumor. It occurs primarily in the uterus and retroperitoneum and accounts for ~24% of soft tissue sarcomas. LMS has been reported to be difficult for clinical management with poor clinical outcome.

So far, several factors or genes have been found to associate with poor outcomes for LMS patients, including DNA aneuploidy (1), vascular invasion (2), *ARL4C* (3, 4) and *c-Myc* expression (5). In particular, high *c-Myc* expression was reported to be an unfavorable prognosis biomarker in terms of overall survival (OS) in LMS (5). *PRUNE2* (prune homolog 2, Drosophila) regulates differentiation, proliferation, and invasiveness of neuroblastoma cell and the up-regulated expression of this protein is a favorable prognosis biomarker in human neuroblastomas (6). Recently, Zhao et al. demonstrated that increased PRUNE2 protein expression is significantly associated with tumor size (*p* = 0.03) and hemorrhage/cyst (*p* = 0.01), and is an independent favorable prognostic factor for OS in LMS (*p* < 0.05) (7). Since identification of prognostic factors will greatly improve cancer diagnosis and treatment, more efforts should be spent on discovering novel prognostic factors for LMS.

With the development in high-throughput sequencing and gene microarray, one way to facilitate the discovery of novel prognostic factors is by studying gene expression profiles of tumors in conjunction with the review of patients' clinical information. However, certain obstacles exist that hinders the use of such data. First, raw microarray datasets are typically buried in the supplementary data files of publications or in the public datasets that are deposited into GEO (8), TCGA or ArrayExpress (9). Second, professionals, who have specialized knowledge and backgrounds to curate the data, are needed to dig into public datasets for prognostic analysis. Third, the clinical follow-up information in the publication or supplied as supporting information is often missing (10). Therefore, user friendly online tools are extremely valuable to provide intuitive curation of these datasets and offer toolkits to quickly visualize and analyze the datasets. Previous studies have reported five pioneer online prognosis analysis tools including KM Plotter (11–14), ITTACA (10), PrognoScan (15), OncoLnc (http://www.oncolnc.org/), and GEPIA (16) for gastric cancer, lung cancer, ovarian cancer, glioma and breast cancer, respectively. However, there is no such tool for LMS. Therefore, an effective analysis tool to access and analyze the prognosis for LMS needs to be established.

In current studies, we have collected public available LMS expression profiling datasets with clinical information and developed a “OSlms” web server, a user-friendly web tool for assessing the relationship between gene expression and LMS patient prognosis using Kaplan Meier plot approach. This web server will help clinicians and basic researchers to determine the prognostic significance of genes of interests and will offer opportunities to identify novel potential important molecules for LMS studies.

## Materials and Methods

### Data Collection

Gene expression profiling datasets of LMS were collected from PubMed, GEO and TCGA (Table 1) according to the following criteria: (1) contains patient follow-up information, (2) includes ≥ 50 LMS samples to enable valid survival analysis, (3) derives from a platform having public probe annotation.

**Table 1:** Dataset source of OSlms.

**Dataset**	**Tissues**	**Array type**	**References**	**No. of samples**
TCGA	LMS	RNASeq	TCGA (17)	105
GSE21050	LMS	HG-U133_Plus_2	Chibon et al. (18)	84
GSE71118	LMS	HG-U133_Plus_2	Lesluyes et al. (19)	79

### Development of OSlms

**Figure 1:**
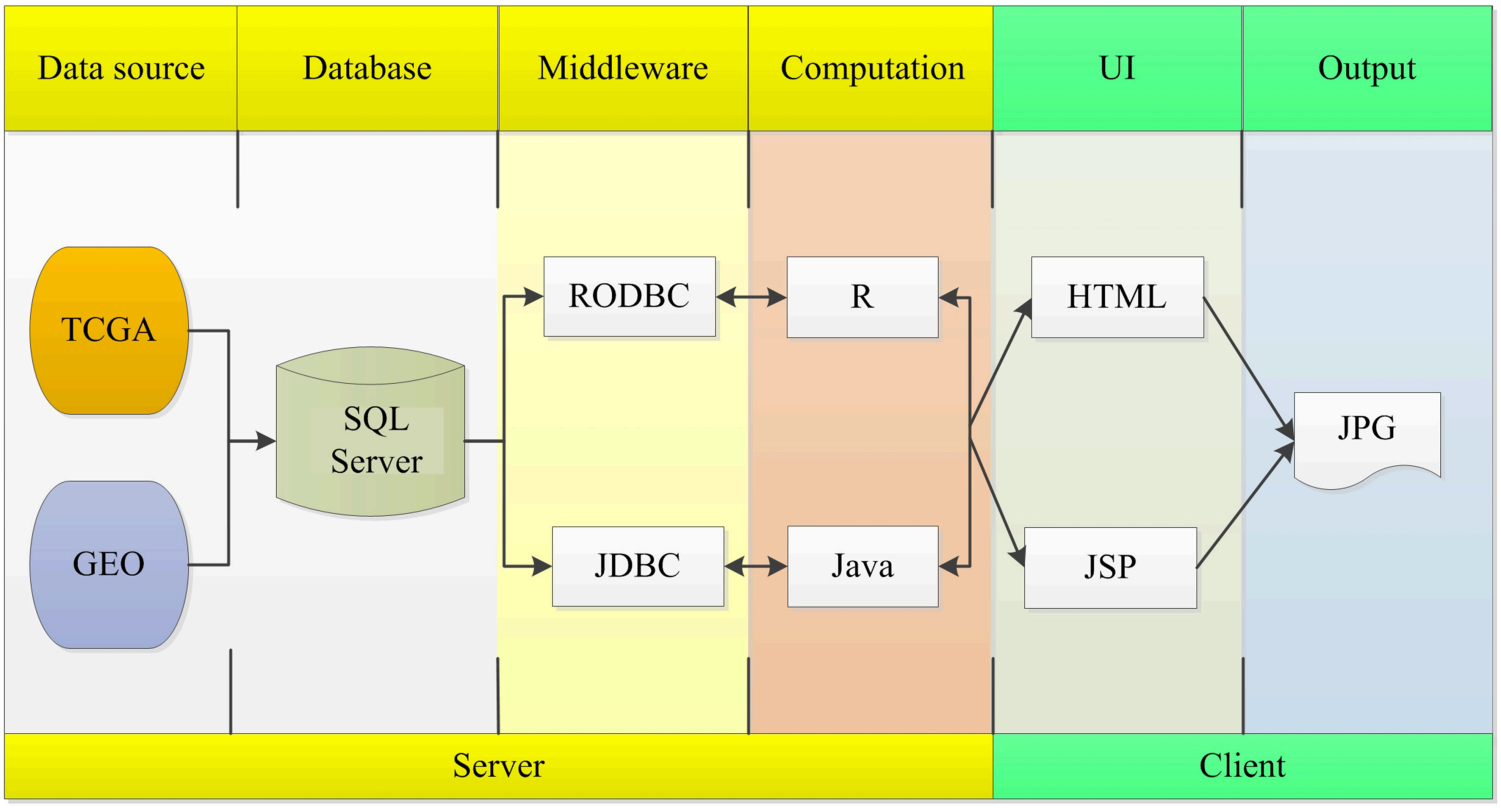
System flow diagram of OSlms.

The OSlms server is deployed in a tomcat server. Front end application was developed in HTML and JSP, which can retrieve user inputs and display the results on the web page. The server application was developed in Java and R, which controls the analysis request and returns the results. The SQL Server database was used to store and manage the gene expression profiles and clinical data. The R package, “RODBC” and “JDBC,” were used as middleware to connect R and SQL Server, and to connect Java and SQL Server. The R packages “survminer” and “survival” generate Kaplan Meier (KM) survival curves and calculates the hazard ratio (HR) with 95% confidence intervals and log rank *p*-value. OSlms can be accessed at http://bioinfo.henu.edu.cn/LMS/LMSList.jsp. System flow diagram is presented in Figure 1.

## Results

### Establishment of the OSlms Web Server

The OSlms web server employs the Kaplan Meier plot to show the association between the investigated gene and survival rate. Prior to the analysis, the users have the options to narrow the analysis in a subgroup of LMS patients based on age, local recurrence status, gender, residual tumor extent and radiotherapy response. Next, when a query is submitted, the expression level of a selected gene will be demonstrated as median, quartile and trichotomy of the expression level. In addition, clinical outcomes, which include overall survival (OS), metastasis free survival (MFS), progression free survival (PFS), disease free interval (DFI), and progression free interval (PFI), can be evaluated based on the selected samples. Finally, prognostic results of selected genes or probe sets targeting selected genes can be output to assist the evaluation of the validity and reliability of a biomarker.

### Functional Validation of Previous Reported Prognostic Biomarkers in the OSlms Web Server

**Figure 2:**
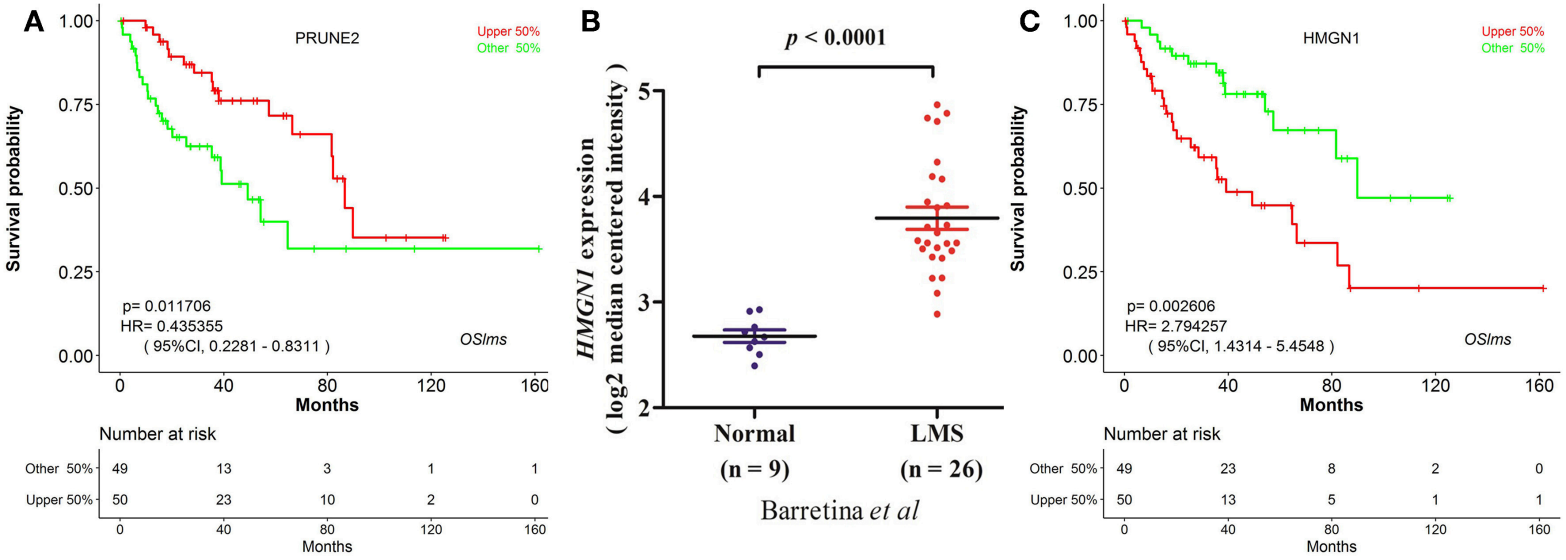
Prognostic test of gene *PRUNE2* and HMGN1 in LMS. **(A)** Kaplan Meier plot for gene *PRUNE2*. The LMS patients with high and low expression levels are designated as red and green colors. Confidence intervals (CI, 95%) and log rank *p*-values are as shown. The X-axis represents survival time and the Y-axis represents survival rate. **(B)** Significant high expression of *HMGN1* was found in LMS (20). **(C)** Kaplan Meier plot for *HMGN1* shows that high (red) and low (green) expression of *HMGN1* is significantly associated with patient survival.

OSlms is a user-friendly tool to assess the prognostic value of genes of interests for LMS patients. As a demonstration, we used the tool to validate the published prognostic biomarker *PRUNE2* (7), and found that LMS patients with high levels of *PRUNE2* have better survival (HR: 0.435, 95%CI: 0.228-0.831, *p* = 0.0117), while patients who exhibited low *PRUNE2* expression have a lower chance of survival (Figure 2A). This result not only supports that elevated *PRUNE2* expression correlates with good prognosis in LMS, but also shows the validity and reliability of this web server.

### Identification of Two Putative New LMS Prognostic Biomarkers in OSlms

To test the predictive power of OSlms, a known prognostic biomarker of lung and breast cancer, *HMGN1* (Alarmin high-mobility group nucleosome-binding protein 1), was analyzed in the OSlms web server. *HMGN1* is a member of the high-mobility group protein family and a non-histone DNA-binding protein that regulates gene transcription and affects chromatin structure by direct binding to nucleosomes (20). In 2015, Wei et al. demonstrated that *HMGN1* is a novel clinical biomarker in early-stage patients of non-small cell lung cancer (21). Meanwhile, Lee et al. showed that *HMGB1* (high mobility group B1) and *HMGN1* are related with tumor-infiltrating lymphocytes in HER2-positive breast cancers (22). Compared with adipose tissue, *HMGN1* mRNA expression was significantly increased in LMS (*p* < 0.0001, Figure 2B) (23). Since the prognostic significance of *HMGN1* in lung cancer and other cancers, we examined the prognostic potency of *HMGN1* for LMS patients. Using OSlms, we found that LMS patients with high *HMGN1* expression have significantly worse OS than the low HMGN1 expression group (HR: 2.794, 95%CI: 1.431-5.455, *p* = 0.0026), suggesting that *HMGN1* could be a novel prognostic indicator of poor overall survival for LMS (Figure 2C).

**Figure 3:**
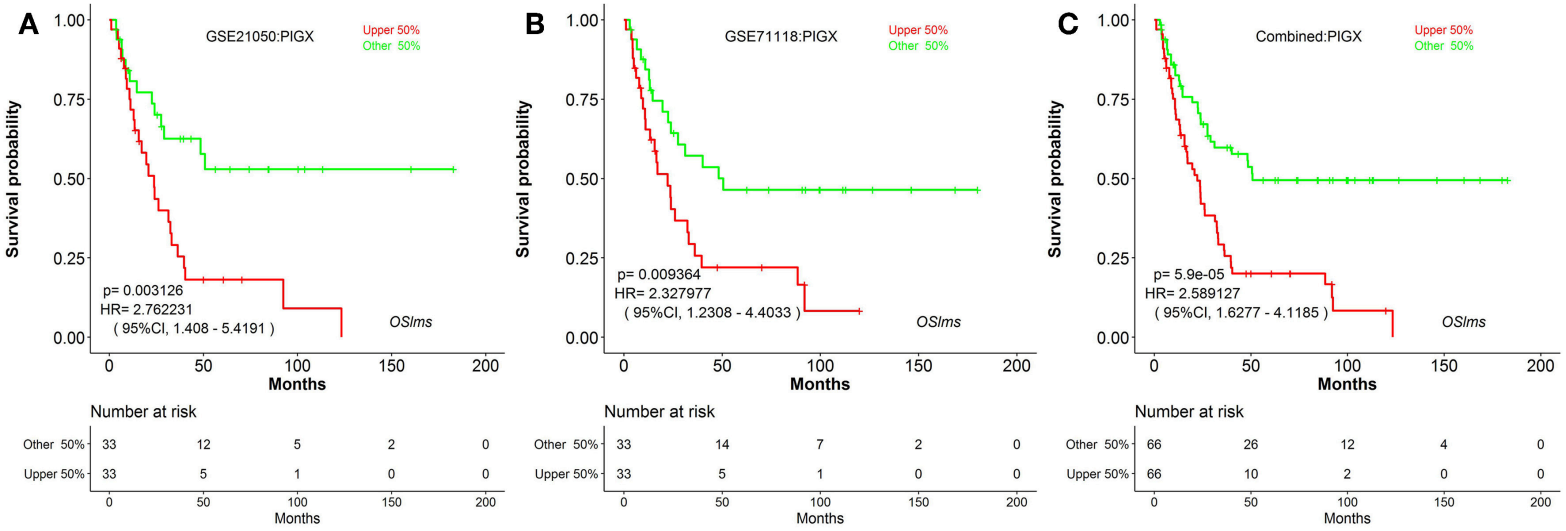
Kaplan Meier plots for high (red) and low (green) *PIGX*-expressing LMS groups in GSE21050 **(A)**, GSE71118 **(B)** and combined datasets **(C)**. Confidence intervals (CI, 95%) and log rank *p*-values are as shown. The X-axis represents survival time and the Y-axis represents survival rate.

To further test the server, another known prognostic biomarker in breast cancer, *PIGX* (Phosphatidylinositol glycan anchor biosynthesis class X), was selected. *PIGX* plays an important role in the biosynthetic pathway of glycosylphosphatidylinositol (GPI)-anchor motif and is found to be highly upregulated in breast cancer cells (24). Nakakido et al. showed that *PIGX* promotes cancer cell proliferation by suppressing putative tumor suppressors, including *EHD2* and *ZIC1* (24). Using OSlms, the high *PIGX* expressing LMS group has significantly worse MFS, while low *PIGX* expressing group has better MFS in both the GSE21050 (HR: 2.762, 95%CI: 1.408–5.419, *p* = 0.0031) and the GSE71118 datasets (HR: 2.328, 95%CI: 1.230–4.403, *p* = 0.0093) (Figures 3A,B). Not surprisingly, in the combined datasets of GSE21050 and GSE71118, high *PIGX* expressing group also showed poor MFS (HR: 2.589, 95%CI: 1.628–4.119, p = 5.9e-05), suggesting that *PIGX* is likely an unfavorable prognostic biomarker for MFS in LMS (Figure 3C).

## Discussion

OSlms is the first online analysis tool to perform prognosis tests and investigations for LMS patient. This online tool provides a platform for clinicians, biologists and other researchers to evaluate the reliability and prognostic significance of genes of interests in LMS. A limitation of this web tool is the patient sample size, for which only 268 samples are currently available in OSlms. When more gene expression datasets of LMS patients become available, we will add new datasets into the OSlms database to improve the power and reliability of this tool.

## Data Availability

Publicly available datasets were analyzed in this study. This data can be found online at: “http://bioinfo.henu.edu.cn/LMS/LMSList.jsp.”

## Author Contributions

XG: study concept and design; LX, YD, XG, XS, TX, ZY, WZ, YW, WL: acquisition of data; QW, LX, YD, XG, XS, TX, ZY, WZ, YW, WL: analysis and interpretation of data. QW, LX, JG, YH, XG: draft of the manuscript; QW, LX, WZ, XG, WL: critical revision of the manuscript for intellectual content.

### Conflict of Interest Statement

The authors declare that the research was conducted in the absence of any commercial or financial relationships that could be construed as a potential conflict of interest.
